# Experiences of maternity care in New South Wales among women with mental health conditions

**DOI:** 10.1186/s12884-020-02972-2

**Published:** 2020-05-11

**Authors:** L. Corscadden, E. J. Callander, S. M. Topp, D. E. Watson

**Affiliations:** 1 Australian Institute of Tropical Health and Medicine, James Cook University, 1 James Cook Dr, Douglas, Queensland 4811 Australia; 2 Bureau of Health Information, Level 11, 67 Albert Avenue, Chatswood, NSW 2067 Australia; 3 School of Medicine, Griffiths University, 170 Kessels Rd, Nathan, QLD 4111 Australia; 4 College of Public Health, Medical & Veterinary Sciences, James Cook University, 1 James Cook Dr, Douglas, Queensland 4811 Australia

**Keywords:** Mental health, Disparities, Patient experiences, Performance

## Abstract

**Background:**

High quality maternity care is increasingly understood to represent a continuum of care. As well as ensuring a positive experience for mothers and families, integrated maternity care is responsive to mental health needs of mothers. The aim of this paper is to summarize differences in women’s experiences of maternity care between women with and without a self-reported mental health condition.

**Methods:**

Secondary analyses of a randomized, stratified sample patient experience survey of 4787 women who gave birth in a New South Wales public hospital in 2017. We focused on 64 measures of experiences of antenatal care, hospital care during and following birth and follow up at home. Experiences covered eight dimensions: overall impressions, emotional support, respect for preferences, information, involvement, physical comfort and continuity. Multivariable logistic regression was used to compare experiences of women with and without a self-reported longstanding mental health condition.

**Results:**

Compared to women without a condition, women with a longstanding mental health condition (*n* = 353) reported significantly less positive experiences by eight percentage points on average, with significant differences on 41 out of 64 measures after adjusting for age, education, language, parity, type of birth and region. Disparities were pronounced for key measures of emotional support (discussion of worries and fears, trust in providers), physical comfort (assistance, pain management) and overall impressions of care. Most women with mental health conditions (75% or more) reported positive experiences for measures related to guidelines for maternity care for women with mental illness (discussion of emotional health, healthy behaviours, weight gain). Their experiences were not significantly different from those of women with no reported conditions.

**Conclusions:**

Women with a mental health condition had significantly less positive experiences of maternity care across all stages of care compared to women with no condition. However, for some measures, including those related to guidelines for maternity care for women with mental illness, there were highly positive ratings and no significant differences between groups. This suggests disparities in experiences of care for women with mental health conditions are not inevitable. More can be done to improve experiences of maternity care for women with mental health conditions.

## Background

High quality maternity care is increasingly understood to represent a continuum of care spanning antenatal, labour and birth and postnatal phases that ensures a positive experience for mothers and families, and responds to their mental health needs. Mental health and maternity care are interconnected. Pregnancy and childbirth is a life changing period for women and their families, with many uncertainties and changes that may bring up new or existing mental health-related needs. It is estimated more than one in 10 mothers experience depressive episodes in the first months following birth [1] with other studies suggesting as many as a quarter of women have depression or other mental disorders in early pregnancy [2]. Mental health issues during and following pregnancy may have serious consequences for both mother and baby [3, 4].

Considering the importance of mental health in relation to the long-term outcomes of mother and child, consideration of mental health has become embedded within maternal health guidelines and policies. In early 2000, the World Health Organization (WHO) set out principles of perinatal care including a focus on ‘women-centred’ care which is culturally appropriate and provides women with information to make informed decisions [5]. In Australia, guidelines for pregnancy care incorporate these WHO principles and highlight guidelines for caring for population groups including women with serious mental illness [6]. In New South Wales (NSW), one aim of the *Towards a Healthy Birth* framework is to develop, implement and evaluate strategies to support women to have a positive experience of pregnancy and birth [7]. At the same time, NSW’s *First 2000 Days Framework* sets out guidelines to help improve opportunities for health intervention between pregnancy and the child’s fifth birthday. It acknowledges the unmet emotional needs of women negatively impact both mothers’ and babies’ outcomes [7].

All women should have a positive pregnancy experience and at-risk women should have experiences responsive to their unique needs, if public health systems aim for high quality, equity and optimal outcomes for all mothers and families. These needs are encompassed in a global push for universal health coverage that ‘leaves no one behind’, and which is often defined equitable coverage of high quality care for all [8]. Despite the recognised importance of people’s experiences with care and guidelines to ensure maternity care is responsive to mental health needs, literature on equity in care experiences has to date, overlooked mental health. Studies from Scotland, England and Italy have explored equity in experiences of maternity care based on large representative patient surveys. These studies have shown women with: lower education [9], from ethnic minority groups [10] younger women, and those from more deprived areas, and those with poorer health, had less positive experiences of care [11]. We have found no study comparing maternity experiences of women with mental health conditions alongside more commonly assessed characteristics such as age, education, and parity.

The aim of this analysis is to answer the following questions:

- Do women with a longstanding mental health condition have more or less positive experiences of care than other mothers?
- Is healthcare responsive to the particular or additional needs of women with mental health conditions from their perspectives?
- Which types of experiences reflect the most pronounced disparities in experiences for mothers with mental health conditions?

## Methods

In 2017, over 62,000 women gave birth in a New South Wales public hospital and all were eligible to participate in the Maternity Care Patient Survey. These women represent a majority of 97,000 deliveries in the state, where the rest occur in private hospitals or out of hospital settings [12]. Three months after birth, a stratified random sample of women in 71 public hospitals with more than 100 deliveries were invited to complete a paper or online survey. The survey excluded women who received inpatient psychiatric care, or who had a stillbirth. Nearly 5000 women over 18 years took part (*n* = 4787, response rate 35%). Women shared their experiences across four stages of antenatal care, care during labour and birth, care in hospital following birth and follow up care at home. The survey is largely based on a survey in England and modified for the Australian context [13] and is available on the Bureau of Health Information’s website (http://www.bhi.nsw.gov.au) along with a technical supplement describing the survey methods, exclusions and representativeness. Responses were weighted to be representative of mothers who gave birth in public hospitals [14].

Mothers were considered as having a long-standing mental health condition based on their response when asked: ‘Which, if any, of the following longstanding conditions do you have?’. The survey lists eight condition options including ‘A mental health condition (eg. depression)’. Women who responded ‘yes’ to that option are referred to in this analysis as having a mental health condition. We interpreted long-standing to mean these women had experienced mental health issues before their most recent pregnancy, whether or not they had an acute episode of illness across the four stages of care.

Bivariate and multivariate analyses were conducted to assess differences in experiences by the presence of a self-reported longstanding mental health condition(s) for 64 measures including prenatal, perinatal and post-natal care. For each survey question, responses were dichotomised to focus on most positive response (‘very good’, ‘always’), with all other response categories combined. Missing and not-applicable responses were excluded. This is consistent with published use of this survey data for public reporting [14]. Analysis of a full set of survey measures is common in other equity focused analyses of survey results [11, 15].

Using logistic regression models, we compared the odds of positive experiences of care between women with and without a self-reported mental health condition for each of the 64 experience measures adjusting for age, remoteness, language spoken at home (English or non-English), parity, birth type and local health district of residence. Covariates were selected on the basis of univariate analyses and literature. In this analysis, differences in experiences that are associated with having a mental health condition, that are not related to other factors such as age, parity or location are referred to as disparities. Disparities are assumed to be amenable to improvement efforts.

In addition, experiences of women with and without mental health conditions were compared by calculating the percentage point difference in reporting the most positive response option. Where a negative percentage point difference suggests women with a mental health condition had less positive experiences.

To summarise responses, survey questions were mapped onto eight dimensions of patient experiences of care based on the literature [16]. These dimensions include:

- overall impressions (e.g. overall ratings, courtesy of doctors),
- emotional support (e.g. discussion of fears, confidence and trust),
- respect for preferences (e.g. treated with respect and involved in decisions),
- information and education (e.g. provided information with clear explanations)
- involvement of family (e.g. family given information and opportunity to talk to professionals)
- physical comfort (e.g. pain management, assistance)
- coordination of care (e.g. organised, appointments on time, no conflicting information)
- continuity and transition (e.g. support in management of condition, know what to do next, told about side effects).

The full list of 64 measures and the domains they were mapped to is provided in Appendix A.

In addition, within the larger list, two sub-groups of questions of interest were identified. First, we identified measures that have been identified in research and practice as being particularly important measures of patient experiences [17–19].

**Table:** 

Overall impressions • Overall, antenatal care was ‘very good’ • Overall, hospital care during birth was ‘very good’ • Overall, hospital care following delivery was ‘very good’ • Overall, follow up care was ‘very good’	Emotional support • Professionals discussed worries and fears during antenatal care • Professionals discussed worries and fears during labour • Had confidence and trust in professionals during labour and birth
Respect for patient preferences • ‘Definitely’ had input about pain relief during labour and birth • ‘Always’ treated with respect and dignity during labour and birth • ‘Definitely’ involved in decisions during labour and birth • ‘Definitely’ involved in decisions about discharge	Information and education • Received enough information about pain relief prior to the birth • Professionals ‘always’ explained antenatal care clearly • Midwives/doctors ‘always’ explained labour and birth clearly • Professionals explained care following birth clearly • Professionals gave enough information self-care after birth
Physical comfort • Professionals did everything to help manage pain after birth	Coordination • Antenatal care was ‘very well organised’ • No conflicting information during labour and birth • No conflicting information about self-care or care for baby

Second, we focused on experience measures that could serve as a proxy for guidelines for maternity care for women with mental illness and, therefore, where experiences would be expected to occur to ensure care is responsive to unique mental health needs. A subset of five questions was identified as related to Australian guidelines for maternal care for women with mental health conditions, including questions about; emotional health, weight gain, and substance use as outlined to follow [6].

**Table:** 

Survey questions	Related guidelines
• Did the health professionals give you advice about the risks of consuming alcohol while pregnant?	As part of providing ‘education about nutrition and ceasing smoking, substance use and alcohol intake in pregnancy’
• Did the health professionals give you advice about the risks of exposure to tobacco smoke while pregnant?	
• During a follow-up appointment, did a midwife or nurse ask you how you were feeling emotionally?	As part of ‘monitoring for early signs of relapse, particularly as medication is often ceased before or during pregnancy’
• Did the health professionals ask you how you were feeling emotionally during your pregnancy?	
• After the birth, did the health professionals give you enough information about how to care for yourself?	

## Results

Almost one in 10 women (7%, *N* = 353) reported they had a mental health condition. These mothers were younger, English speaking, born in Australia, have less formal education, and reside in rural areas (Table 1). The factors associated with overall ratings of care at each stage of care are presented in Table 2.

**Table 1:** Characteristics of the respondents, by mental health condition group

Percentage (Number of respondents)		Mental health group (*n* = 353)	No condition group (*n* = 4315)	Total (*n* = 4668)	
**Age**	18–24	16% (62)	7% (339)	8% (401)	*p* < 0.001
	25–29	22% (88)	24% (1142)	24% (1230)	
	30–34	38% (127)	40% (1682)	40% (1809)	
	35+	24% (76)	29% (1152)	28% (1228)	
**Language spoken at home**	English	93% (338)	72% (3540)	74% (3878)	*p* < 0.001
	Non-English	7% (14)	28% (749)	26% (763)	
**Born in Australia**	Yes	83% (311)	55% (2926)	57% (3237)	*p* < 0.001
	No	17% (42)	45% (1389)	43% (1431)	
**Education**	Post graduate/higher degree	13% (49)	19% (718)	19% (767)	*p* < 0.001
	Trade or technical certificate	26% (98)	24% (1089)	24% (1187)	
	University degree	23% (73)	34% (1439)	34% (1512)	
	Completed Year 12	23% (73)	14% (643)	14% (716)	
	Less than Year 12	14% (60)	9% (415)	9% (475)	
**Socioeconomic status of postal code area**	Quintile 1: Most disadvantaged	17% (68)	19% (751)	19% (819)	*p* = 0.676
	Quintile 2	15% (82)	17% (997)	17% (1079)	
	Quintile 3	22% (85)	24% (1028)	24% (1113)	
	Quintile 4	26% (69)	22% (816)	22% (885)	
	Quintile 5: Least disadvantaged	19% (49)	18% (720)	18% (769)	
Rurality*	Major cities	65% (140)	79% (2282)	78% (2422)	*p* < 0.001
	Inner regional	27% (149)	16% (1418)	17% (1567)	
	Outer regional, remote	8% (64)	5% (614)	5% (678)	
**Birth type**	Vaginal birth	56% (206)	59% (2637)	59% (2843)	*p* = 0.609
	Assisted vaginal birth	17% (47)	13% (498)	14% (545)	
	Caesarean section (emergency)	14% (51)	14% (591)	14% (642)	
	Caesarean section (planned)	13% (48)	14% (568)	14% (616)	
**Induced**	No	59% (188)	57% (2252)	57% (2440)	*p* = 0.609
	Yes	41% (116)	43% (1442)	43% (1558)	
**Given birth before (parity)**	No	52% (183)	48% (1995)	49% (2178)	*p* = 0.336
	Yes	48% (170)	52% (2317)	51% (2487)	
**Hospital size**	Large and specialist	48% (63)	54% (1035)	54% (1098)	*p* = 0.088
	Major	40% (153)	36% (1718)	36% (1871)	
	Small	13% (137)	10% (1562)	10% (1699)	
**Provider of most antenatal care**	Midwife(s)	62% (202)	61% (2542)	61% (2744)	*p* = 0.786
	Obstetrician	16% (52)	17% (657)	17% (709)	
	GP	14% (71)	16% (850)	15% (921)	
	Other	7% (28)	6% (266)	6% (294)	

The distribution of characteristics for women with a mental health condition are significantly different from the characteristics of those without a mental health condition (*p* ChiSq< 0.001). Of all respondents, 7% self-reported a longstanding mental health condition. Missing responses were excluded

**Table 2:** Percentage of women rating care as ‘very good’ by stage of care and patient characteristics

		Antenatal	Labour and birth	Hospital following birth	Follow up at home
New South Wales (NSW)		63	75	60	67
Age	18–24	**53 L**	**64 L**	**50 L**	**61 L**
	25–29	64	74	58	66
	30–34	63	77	62	69
	35+	65	76	62	68
Immigrant	Born in Australia	66	77	60	68
	Not born in Australia	59	72	60	66
Language spoken at home	Non-English	65	77	60	68
	English	57	70	59	66
Longstanding mental health condition	No	64	76	60	68
	Yes	**55 L**	**63 L**	**52 L**	**60 L**
Socio-economic status of area	Quintile 1: Most disadvantaged	58	68	59	66
	Quintile 2	60	76	60	63
	Quintile 3	65	75	58	**72H**
	Quintile 4	63	75	60	66
	Quintile 5: Least disadvantaged	**70H**	**81H**	63	69
Rurality	Major cities	62	74	59	68
	Inner regional	67	76	62	66
	Outer regional and remote	69	80	**67H**	61
Education	Post graduate/higher degree	66	76	60	68
	University degree	62	74	59	67
	Trade or technical certificate	63	77	61	67
	Completed Year 12 or equivalent	62	72	60	66
	Less than Year 12 or equivalent	62	77	59	69
Survey mode	Hardcopy	64	76	60	68
	Online	61	73	60	67
Baby spent time in intensive care	No	64	77	61	68
	Yes	58	67	57	63
Type of birth	Assisted vaginal birth	59	69	57	67
	Caesarean section (emergency)	59	67	56	62
	Caesarean section	62	78	65	68
	Vaginal birth	65	78	60	69
Induced	No	65	76	61	69
	Yes	61	73	57	66
Given birth before (parity)	No	63	73	56	64
	Yes	64	77	63	**71H**
Who provided most antenatal care	Midwives	66	77	61	70
	Obstetrician	61	72	52	61
	GP	56	72	63	65
Hospital size	Large, or specialist hospitals	61	75	59	67
	Major hospitals	63	73	59	68
	Smaller district hospitals	**74H**	**82H**	**72H**	67

Descriptive results where ‘H’ denotes the highest two ratings in a column and “L” denotes the lowest two ratings

Overall, 63% of women rated their antenatal care as ‘very good’ – this ranged from lows of 53% among women aged 18 to 24, and 55% among women with a mental health condition, to 74% of women from small hospitals. Similarly, for care during labour and birth and hospital care following birth, younger mothers and those who had mental health conditions were less likely to report high ratings. For follow up care at home, women from rural areas, giving birth in small hospitals, and those from high socio-economic status areas were more likely to rate the care received as ‘very good’ (Table 2).

### Comparing differences in experiences for selected experience measures

**Fig. 1:**
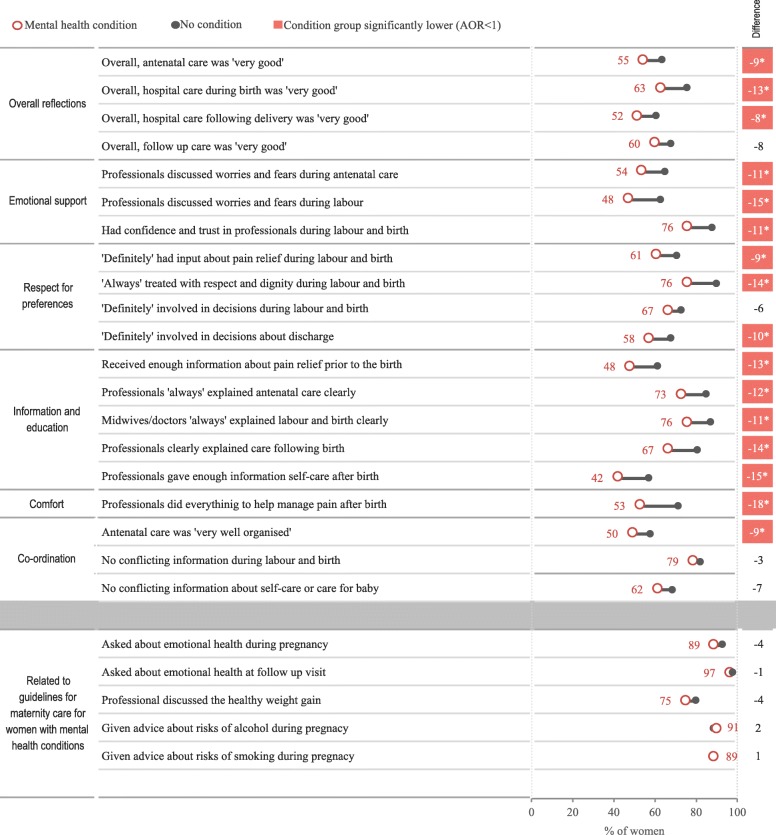
Percentage reporting the most positive response for selected questions by condition group. Notes: Selected questions include experiences that matter most (20 questions) or related to guidelines (65 questions). Women without a mental health condition are the reference group, such that negative percentage point difference values represent less positive experiences for women with a mental health condition. Percentage point differences are shaded were care experiences are statistically significantly less positive for women with mental health conditions after adjusting for age, language, education, parity, birth type, and local health district

To determine if women with longstanding mental health condition(s) have more or less positive experiences than other mothers - experience measures were compared between women with and without a mental health condition adjusting for age, language, education, parity, type of birth, and local health district (Fig. 1).

Across commonly reported measures of experiences, women with mental health conditions reported significantly less positive experiences for 16 of 20 measures (top part of Fig. 1). For example, 76% of women with a mental health condition reported that they were ‘always’ being treated with respect, compared to 90% of women with no condition. Further, fewer than half of women with a mental health condition (48%) said they received enough information on pain relief, compared to 62% of women with no condition. Similarly, fewer than half of women with a mental health condition (48%) reported that they discussed worries and fears, compared to 63% of women with no condition.

Women with mental health conditions offered high ratings of care for measures related to guidelines for maternity care for women with mental illness. There were no significant differences between women with and without mental health conditions across six experience measures selected to serve as a proxy for guidelines (bottom of Fig. 1). About nine in 10 women in both groups were asked how they were feeling emotionally during antennal check-ups – 89% of those with a mental health condition and 93% of women with no condition. Nearly all women said they were asked how they were feeling emotionally during follow-up care (97 and 98%).

### Comparing differences across domains and stages of care

To assess disparities in experiences across domains and stages of care, Table 3 summaries differences between women with and without mental health conditions across 64 measures. The number of measures where there were significant differences (Table 3a) and the percentage-point differences between groups are presented across eight domains and four stages of care (Table 3b).

**Table 3:** Number of significant disparities, and average difference in experiences by dimension and stage of care

Experience domain	Stage of maternity care				
	Antenatal (17 questions)	During birth (18 questions)	Hospital following birth (23 questions)	Follow up at home (6 questions)	Total (64 questions)
a) ***Number of measures where there were significant disparities for women with mental health conditions***^a^					
Overall impression	1	2	4		7 of 9
Emotional support	2	2	1		5 of 7
Physical comfort		2	6		8 of 9
Information and education	2	1	3		6 of 11
Involvement of friends and family		1	1		2 of 2
Respect for patient preferences		4	2	2	8 of 11
Continuity and transition			1		1 of 3
Coordination of care	2	1	1		4 of 12
Total (64 questions)	7	13	19	2	41 of 64
b) ***Average percentage point difference between women with and without a mental health condition***					
Overall impression	-7	−10	−12	− 8	− 10
Emotional support	− 9	−13	− 13	−1	− 10
Physical comfort		−13	−10		−11
Information and education	−5	−11	−12	2	−8
Involvement of friends/ family		−9	− 8		−8
Respect for patient preferences		−7	−9	−8	−7
Continuity and transition			−10	2	−6
Coordination of care	−2	−4	−9		−4
Total (64 questions)	−5	−8	−11	−3	− 8

Note: Women without a mental health condition are the reference group, such that negative percentage point difference values represent less positive experiences for women with a mental health condition. Percentage point differences are counted where care experiences are significantly **less positive** for women with mental health conditions after adjusting for age, language, education, parity, birth type, and local health districts

^a^Women with mental health conditions had significantly more positive experiences on only one measure for information and education, at follow up at home about safe sleeping for the baby, and is not noted in this table counts of significant disparities

See Appendix A for results for all 64 measures

Women with mental health conditions had significantly less positive experiences for 41 of 64 measures, and only more positive on one measure. For example, women with mental health conditions were less positive across seven of nine overall experience measures – including overall ratings of antenatal care, care during labour, care in hospital, and measures about the kindness of health professionals during and following birth. Across all stages of maternity care, experiences were significantly less positive for women with mental health conditions for 7 of 17 antenatal measures, 13 of 18 birth-related measures, 19 of 23 measures about hospital care following birth, and 2 of 6 measures of follow up at home. Experiences were significantly more positive for one measure (i.e. information on safe sleeping) (Table 3a). In relation to the magnitude of differences between women with and without mental health conditions, the percentage point differences were most pronounced during hospital care following birth, with an average adjusted difference of − 11. There were differences of 8 percentage points or more on average across all dimensions of care in that stage (Table 3b).

Across experience dimensions, the differences between women with and without a mental health condition, were most pronounced for experiences about comfort (average percentage point difference: − 11) and overall impressions of care and emotional support (average percentage point difference was − 10 for both measures). For example, 76% of women with a mental health condition said they ‘always’ had confidence and trust in doctors and midwives providing care during labour, compared to 88% of women with no condition (percentage point difference: − 12) (Table 3b). Results for all 64 experience measures are available in the Technical Appendix.

## Discussion

A majority of women reported positive overall ratings of antenatal care, care during labour and birth, hospital care following birth and follow up care at home. In particular, women provided high ratings to experience measures related to guidelines for maternity care related to mental illness. Australian guidelines that can be measured from mothers’ perspectives include, provision of information about how to care for themselves if they need it (e.g. provide psychoeducation, advise about the benefits of support groups, advice on benefits of counselling), monitoring weight gain and substance use for example. There were no significant differences for these measures between women with and without a self-reported mental health condition. This confirmed our hypothesis that women with mental health conditions would have the same or ideally more positive experiences on measures related to these guidelines. Australian guidelines recommend women are asked about their emotional health [6]. About nine in 10 women both with and without mental health conditions said they were asked about emotional health during antennal check-ups (89 and 93%), follow-up care (97 and 98%). Internationally, most women recalled being asked about their emotional health during pregnancy (82%) and in the postnatal period (90%) [20]. However, this is the first study we are aware of to look at questions related to emotional well-being by mental health condition group.

While women who have a mental health condition reported positive experiences related to guidelines, they reported less positive experiences with care across all eight experiences domains and all four stages of care compared to women with no condition. Women with mental health conditions were less positive about experiences for 41 of 64 measures, and only more positive on one, after adjusting for age, language and birth type among other factors. Disparities for the mental health group were most pronounced for experience dimensions related to comfort, emotional support, respect for preferences, and overall reflections of care. Across stages of maternity care, differences were most pronounced during hospital care following birth. In contrast, for questions about continuity and coordination, and care during antenatal and follow-up stages of care there were fewer significant differences. Consistent with this finding, analysis of maternity care in Scotland showed that women reporting poorer health, also reported less positive experiences in most domains of care including pain relief, communication, involvement in decisions, confidence and overall ratings [11].

A New South Wales Ministry of health report notes, “Preparing mothers emotionally for birth, and promoting the mental health of parents and carers in pregnancy, can make a dramatic difference to how parents and carers experience birth, and how they cope in their transition from pregnancy to parenthood” [7]. This analysis brings to light several areas of women’s experiences of maternity care where care could be improved for mothers with mental health conditions. Findings build on a state report that showed women in New South Wales with mental health conditions had less positive experiences of care than those with no condition for a subset of measures [17]. While women offer positive ratings of maternal care and very high ratings on experience measures identified as proxies for guidelines, women who report having mental health conditions offer less positive ratings on many domains of care critically important to clinical quality and outcomes including, for example, emotional support, respect for preferences, information and education and pain management.

This study adds to the evidence that despite universal care in Australia, vulnerable women do not experience the same complete and quality access to maternity care that has been posed elsewhere [21]. The current study suggests that women with mental health conditions which may be among those most in need of support during and following pregnancy, but are often less likely to get it. This was also true for experiences related to emotional support, where it would be expected that those with mental health conditions should report better experiences than those with no mental health conditions. Where other studies on disparities in experiences have focused on young mothers, from diverse or low-income backgrounds [11], this current study demonstrates that mothers with mental health conditions need more support in terms of overall maternal care and care for mental health needs.

Ensuring women with mental health-related needs get access to mental health care during and after their pregnancy is a part of providing good continuity of care. There is evidence that some models of care, such as those providing continuity of care, can have benefits particularly for vulnerable groups [22]. However, there are barriers that may prevent women from seeking mental health treatment include; stigma, a fear of losing parental rights, negative experiences with health professionals, and a perceived lack of skills among professionals to help them [23]. Professionals themselves may not feel prepared to deal with mental health needs. A study of midwives suggests they do not feel well equipped to deal with mental health issues [24]. In this study, it was not possible to consider how possible differences in staff or service settings may enable patient-provider relationships or responsive care to mothers with mental health conditions.

Maternity experience surveys and monitoring experiences for mothers from vulnerable groups may help draw attention to these needs more regularly and show where they differ regionally. In Australia, indicators and regular reporting on maternity care exclude experiences and do not focus on intersections with mental health [25]. Monitoring, transparency and regular reporting is important to help the public and providers understand the variation in experiences. Further, data linkage between guideline related experiences and outcomes could help to substantiate the degree to which better experiences lead to better outcomes. This may help improve the evidence base to include patient experiences as part of practice guidelines.

### Limitations

This secondary analysis of cross-sectional data cannot be used to determine if the mental health condition was present before birth or would have been considered only after birth. The survey likely under-represents mothers with serious mental health conditions as the survey excludes women who spent any time in a psychiatric unit, had a history of self-harm or expressed suicide ideation. There were too few Aboriginal women represented in the survey to consider their experiences as a group in this analysis. Other analysis suggests that some minority groups are not included due to lower representation [11], and postal or paper surveys may not be the right method to capture their experiences. The survey response rate of 35% may also introduce sources of bias that were not possible to determine from a review of the evidence of representativeness outlined in the survey technical supplement.

Analysis findings may be sensitive to methods used. There are different findings in the literature on the association between experience and age or parity and experience for example. In addition, the analysis of the most positive category may not be sensitive to the fact that some groups may have reporting tendencies that avoid extreme responses.

With survey data alone it is not possible to establish meaningful differences in experiences. Using linked data in future analysis may help show possible clinical significance of different experiences of care. However, in this analysis the focus is an aspirational goal of ensuring all women have positive experiences.

## Conclusions

Women with mental health conditions offer high ratings on a small selection of experience measures identified as proxies for guidelines, but otherwise report substantially less positive experiences of care than women without conditions across all domains and stages of the maternal care journey. They represent a unique and important population group to consider who have particular needs that must be better understood and addresses. Hospital care following birth, emotional support and respect for patient preferences are key areas for improvement of experiences for women with mental health conditions, where there were pronounced disparities. There are also opportunities for better data collection and monitoring of experiences of screening for mental health issues. This would help to determine if women who identify that they are in need of help with mental health conditions are having their needs met.

## Data Availability

More information about the data source can be found at http://www.bhi.nsw.gov.au/nsw_patient_survey_program/maternity_care_survey. Data is currently only available to employees of the Bureau of Health Information (including two of the co-authors); Data requests can be made http://www.bhi.nsw.gov.au/About_us/contact_us. No licences were required or acquired to access the data.

## Appendix

### Technical Appendix A

**Table 4:** Percentages, difference and adjusted odds of reporting positive experiences, women with compared to those without, a mental health condition

Dimension	Stage	Question	Response	MHC (n)	MHC (%)	No MHC (%)	GAP	AOR	AOR 95% CI
Overall	1	#Overall, how would you rate the antenatal care you received during your pregnancy?	Very good	341	55	64	**−9***	0.69	(0.51,0.94)
Overall	1	Were the health professionals providing your antenatal care polite and courteous?	Yes, always	345	84	90	−6	0.67	(0.43,1.05)
Overall	2	#Overall, how would you rate the care you received in the hospital during your labour and birth?	Very good	351	63	76	**−13***	0.55	(0.4,0.76)
Overall	2	Were the midwives or doctors kind and caring towards you? [during labour and birth]	Yes, always	352	82	89	**−7***	0.58	(0.39,0.86)
Overall	3	#Overall, how would you rate the care you received in the hospital after your baby was born?	Very good	350	52	60	**−8***	0.72	(0.53,0.97)
Overall	3	If friends and family asked about your maternity experience at the hospital where you gave birth, how would you respond?	Would speak highly	352	70	81	**−11***	0.57	(0.41,0.8)
Overall	3	After the birth of your baby, were the health professionals taking care of you kind and caring?	Yes, always	352	63	78	**−14***	0.5	(0.36,0.7)
Overall	3	Looking back, do you feel that the length of your stay in hospital was …?	About right	352	71	84	**− 13***	0.49	(0.34,0.69)
Overall	4	#Overall, how would you rate the care you received in the first two weeks after arriving home from the hospital?	Very good	353	60	68	−8	0.75	(0.55,1.02)
Emotional	1	(MH) Did the health professionals ask you how you were feeling emotionally during your pregnancy?	Yes	334	89	93	−4	0.67	(0.4,1.11)
Emotional	1	#Did the health professionals discuss your worries or fears with you? [at antenatal check-ups]	Yes, completely	240	54	65	**−11***	0.67	(0.46,0.97)
Emotional	1	Did you have confidence and trust in the health professionals providing your antenatal care?	Yes, always	345	71	83	**−11***	0.54	(0.39,0.76)
Emotional	2	#Did you have confidence and trust in the midwives or doctors taking care of you during your labour and birth?	Yes, always	352	76	88	**−11***	0.48	(0.33,0.69)
Emotional	2	#Did a midwife or doctor discuss your worries or fears with you? [during labour and birth]	Yes, completely	214	48	63	**−15***	0.54	(0.37,0.78)
Emotional	3	Shortly after the birth, did a health professional talk to you about how the birth had gone?	Yes	353	62	75	**−13***	0.55	(0.41,0.76)
Emotional	4	(MH) During a follow-up appointment, did a midwife or nurse ask you how you were feeling emotionally?	Yes	337	97	98	−1	0.63	(0.29,1.39)
Information	1	(MH) Did the health professionals give you advice about the risks of consuming alcohol while pregnant?	Yes	324	91	89	2	1.19	(0.68,2.09)
Information	1	(MH) Did the health professionals give you advice about the risks of exposure to tobacco smoke while pregnant?	Yes	329	89	88	1	1.04	(0.61,1.75)
Information	1	(MH) Did the health professionals discuss the importance of healthy weight gain with you? [at antenatal check-ups]	Yes	321	75	80	−4	0.77	(0.53,1.11)
Information	1	#Did the health professionals providing your antenatal care explain things in a way you could understand?	Yes, always	346	73	85	**−12***	0.51	(0.35,0.74)
Information	1	#Did you receive enough information about pain relief options prior to the birth?	Yes, definitely	314	48	62	**−13***	0.58	(0.42,0.8)
Information	2	#During your labour and birth, did the midwives or doctors explain things in a way you could understand?	Yes, always	352	76	87	**−11***	0.49	(0.34,0.71)
Information	3	#(MH) After the birth, did the health professionals give you enough information about how to care for yourself?	Yes, completely	344	42	57	**−15***	0.56	(0.41,0.75)
Information	3	#After the birth of your baby, did the health professionals explain things in a way you could understand?	Yes, always	352	67	81	**−14***	0.51	(0.37,0.71)
Information	3	After the birth, did the health professionals give you enough information about how to care for your baby?	Yes, completely	304	38	56	**−18***	0.48	(0.35,0.66)
Information	3	Did midwives in the hospital work with you to show you a good position for breastfeeding your baby?	Yes	305	88	91	−3	0.74	(0.43,1.29)
Information	4	At any point during your pregnancy or after the birth, were you shown or given information about safe sleeping for your baby?	Yes	343	99	96	2*	2.69	(1.27,5.7)
Respect	2	#Were you involved, as much as you wanted to be, in decisions during your labour and birth?	Yes, definitely	346	67	73	−6	0.76	(0.56,1.04)
Respect	2	#Did you feel you were treated with respect and dignity during your labour and birth?	Yes, always	352	76	90	**−14***	0.37	(0.25,0.54)
Respect	2	During your labour, were you able to move around and choose the position that made you most comfortable?	Yes, most of the time	280	60	67	**−7***	0.7	(0.49,0.99)
Respect	2	Were you offered the option of being in a bath during labour?	Yes	226	50	53	−3	0.85	(0.58,1.25)
Respect	2	#Did you have enough say about your pain relief during your labour and birth?	Yes, definitely	351	61	71	**−9***	0.66	(0.49,0.89)
Respect	2	Were you given enough privacy in the birth room or theatre?	Yes, always	351	84	90	**−6***	0.62	(0.41,0.93)
Respect	2	Did you have skin to skin contact with your baby shortly after the birth?	Yes	308	97	96	0	1.04	(0.46,2.35)
Respect	3	#Did you feel involved in decisions about your discharge from hospital?	Yes, definitely	349	58	68	**−10***	0.62	(0.46,0.85)
Respect	3	Were your decisions about how you wanted to feed your baby respected by the health professionals?	Yes, always	347	74	82	**−7***	0.66	(0.46,0.95)
Respect	4	In general, did you feel that the midwife or nurse listened to you? [at follow-up appointment]	Yes, always	342	83	92	**−9***	0.48	(0.31,0.74)
Respect	4	In general, did you have enough time with the midwife or nurse to ask questions or discuss any concerns? [at follow-up]	Yes, definitely	342	84	91	**−7***	0.55	(0.35,0.86)
Involvement	2	During your labour and birth, was your birthing companion involved as much as they wanted to be?	Yes, definitely	347	79	88	**−9***	0.56	(0.38,0.82)
Involvement	3	Were the visiting times convenient for your friends and family?	Yes, definitely	340	66	74	**−8***	0.66	(0.47,0.92)
Comfort	2	Do you think the midwives or doctors did everything reasonable to help you manage your pain during your labour and birth?	Yes, definitely	350	66	77	**−11***	0.57	(0.42,0.79)
Comfort	2	Were you able to get assistance from midwives or doctors when you needed it? [during labour and birth]	Yes, always	345	67	83	**−16***	0.43	(0.31,0.61)
Comfort	3	#Do you think the health professionals did everything they could to help you manage your pain after the birth of your baby?	Yes, definitely	274	53	71	**−18***	0.46	(0.33,0.65)
Comfort	3	After the birth of your baby, were you able to get assistance or advice from health professionals when you needed it?	Yes, always	351	54	70	**−16***	0.51	(0.37,0.68)
Comfort	3	How clean were the wards or rooms you stayed in after the birth of your baby?	Very clean	350	63	70	**−7***	0.7	(0.51,0.97)
Comfort	3	How clean were the toilets and bathrooms you used after the birth of your baby?	Very clean	351	60	67	**−7***	0.69	(0.51,0.95)
Comfort	3	Bothered by noise, lack of privacy, lack of security or lighting during stay in hospital	Not bothered	349	38	44	**−7***	0.71	(0.52,0.95)
Comfort	3	How would you rate the hospital food?	Very good	344	14	17	−3	0.78	(0.51,1.19)
Comfort	3	Did the hospital provide access to food when you needed it?	Yes, always	330	49	61	**−12***	0.62	(0.46,0.85)
Coordination	1	#How well organised was the antenatal care you received at your check-ups?	Very well organised	345	50	58	**−9***	0.71	(0.53,0.95)
Coordination	1	How many weeks pregnant were you when you had your first appointment for antenatal care?	Less than 14 weeks	339	43	40	3	1.16	(0.85,1.57)
Coordination	1	How much of this time did you usually spend waiting to be seen? [antenatal]	Under 30 min	345	54	55	−1	0.86	(0.64,1.16)
Coordination	1	Do you think the amount of time you waited was...? [antenatal]	About right	342	57	56	0	0.94	(0.69,1.27)
Coordination	1	Was there any time when the health professionals needed access to your medical history and it was not available? [antenatal]	No	344	67	75	**−8***	0.66	(0.48,0.92)
Coordination	1	Were you provided with a personal antenatal card, where information about your antenatal check-ups was recorded?	Yes	339	94	96	−3	0.6	(0.31,1.15)
Coordination	1	Did the health professionals update your personal antenatal card at every check-up?	Yes	316	98	97	1	1.47	(0.76,2.85)
Coordination	2	#Did midwives or doctors ever give you conflicting information during your labour and birth?	No	351	79	82	−3	0.83	(0.56,1.22)
Coordination	2	Had you previously met any of the midwives or doctors who cared for you during your labour and birth?	Yes	348	49	49	0	0.92	(0.68,1.25)
Coordination	2	Did the midwives or doctors who you did not already know, introduce themselves to you during your labour and birth?	Yes, always	327	76	85	**−9***	0.54	(0.37,0.79)
Coordination	3	#After the birth, did you ever receive conflicting information from health professionals about how to care for yourself/your baby?	No	353	62	69	−7	0.76	(0.56,1.03)
Coordination	3	Did you ever receive conflicting advice about feeding your baby from the health professionals?	No	346	57	69	**−12***	0.64	(0.47,0.87)
Continuity	3	Before leaving hospital, were you given enough information about caring for yourself and your baby at home?	Yes, completely	335	45	64	**−19***	0.45	(0.34,0.61)
Continuity	3	Did hospital staff tell you who to contact if you were worried about your health or your baby’s health after you left hospital?	Yes	335	93	95	−2	0.8	(0.41,1.56)
Continuity	4	In the first 2 weeks after arriving home, had a follow-up appointment with a midwife or nurse?	Yes at home	352	89	86	2	1.28	(0.85,1.93)

Adjusted Odds Ratio (AOR) of positive experience for women with compared without a mental health condition after adjusting for age, language, education, type of birth, parity, and local health district of residence are denoted by “*” where significant at *p* < 0.05. Stage 1: Antenatal, stage 2: Labour and birth, stage 3: hospital care following birth, stage 4: follow up care. Mental health condition group (MHC), no condition group (no MHC), Gap = percentage point difference MHC group minus no MHC group results based on *unrounded values*. Question text denoted by (MH) were questions identified as proxy measures related to guidelines for maternity care for women with mental illness, and those denoted by “#” are considered as more important measures of experience
